# Lipid Accumulation in Host Cells Promotes SARS-CoV-2 Replication

**DOI:** 10.3390/v15041026

**Published:** 2023-04-21

**Authors:** Tatjana Seitz, Christian Setz, Pia Rauch, Ulrich Schubert, Claus Hellerbrand

**Affiliations:** 1 Institute of Biochemistry, Friedrich-Alexander-Universität Erlangen-Nürnberg, D-91054 Erlangen, Germany; tatjana.seitz@fau.de; 2 Institute of Virology, Friedrich-Alexander-Universität Erlangen-Nürnberg (FAU), D-91054 Erlangen, Germany; christian.setz@uk-erlangen.de (C.S.);

**Keywords:** SARS-CoV-2, COVID-19, fatty acid, obesity, hyperlipidemia, coronavirus, variant of concern delta

## Abstract

Coronavirus disease-19 (COVID-19) is still affecting the lives of people around the globe and remains a major public health threat. Lipid levels in the host cells have been shown to promote SARS-CoV-2 replication, and since the start of COVID-19 pandemic, several studies have linked obesity and other components of the metabolic syndrome with severity of illness, as well as mortality in patients with COVID-19. The aim of this study was to obtain insights into the pathophysiological mechanisms of these associations. First, we established an in vitro model simulating high fatty acid levels and showed that this situation induced the uptake of fatty acids and triglyceride accumulation in human Calu-3 lung cells. Importantly, we found that lipid accumulation significantly enhanced the replication of SARS-CoV-2 Wuhan type or the variant of concern, Delta, in Calu-3 cells. In summary, these findings indicate that hyperlipidemia as found in patients with obesity promotes viral replication and herewith the disease course of COVID-19.

## 1. Introduction

The pandemic of coronavirus disease 2019 (COVID-19), caused by the severe acute respiratory syndrome coronavirus 2 (SARS-CoV-2), has resulted in considerable morbidity and mortality throughout the globe, with significant persistent symptoms [1]. Ongoing waves and the emergence of new SARS-CoV-2 variants have posed significant health concerns and socio-economic threats to the world.

Different studies have shown that patients with obesity and other components of the metabolic syndrome are the most susceptible and are more prone to severe side effects when affected by this virus. In France, obesity was identified as an independent risk factor for SARS-CoV-2 infection, and the proportion of patients who required invasive mechanical ventilation increased with BMI [2]. In Spain, obesity was the strongest comorbidity among patients admitted to intensive care units (ICU) [3]. In the UK, a report from the Intensive Care National Audit and Research Center (ICNARC) showed that of the 9272 COVID-19 patients admitted to critical care units in 2020, 39.3% were obese and 35% were overweight [4]. In a retrospective analysis of COVID-19 patients admitted to a hospital in Wuhan, China, 88% of non-survivor patients had a BMI > 25 kg/m^2^ [5]. A cross-sectional study from 50,402 COVID-19 patients in Germany showed that obesity and lipid metabolism disorders represent age-independent risk factors for the development of long COVID syndrome [6]. Together, there is a clear indication that metabolic alterations determine the risk for unfavorable disease courses along all phases of COVID-19. Still, the pathophysiological mechanisms for these notions are only partially understood.

Several lines of evidence suggest that lipid metabolism alterations in cells are critically involved in the pathogenicity of SARS-CoV-2. Nardacci et al., demonstrated accumulation of lipid droplets in the lungs of deceased COVID-19 patients, as well as in SARS-CoV-2-infected Vero E6 cells [7]. In line with this, a study by Dias et al. revealed that monocytes derived from COVID-19 patients exhibit abundant lipid droplets compared to monocytes from healthy donors [8]. Furthermore, SARS-CoV-2 infection was shown to trigger lipid droplet formation in the human lung epithelial cell lines A549 and Calu-3, as well as in the human lung microvascular endothelial cell line HMVEC-L [8,9]. Interestingly, Dias et al. identified colocalization of viral particles with lipid droplets in SARS-CoV-2 infected Vero cells, suggesting that lipid droplets might act as an assembly platform [8].

Conversely, fatty acids can also negatively affect SARS-CoV-2 infectivity, making the situation even more complex. As reviewed by Toelzer et al., linoleic acid stabilizes the spike protein of the virus to a non-infectious conformation and thereby prevents binding to the host cell, suggesting linoleic acid supplementation as a beneficial strategy in the early course of SARS-CoV-2 infection [10]. Furthermore, linoleic acid, oleic acid, and arachidonic acid have been shown to reduce attachment to human cells [11] and fatty acid supplementation was found to ameliorate outcomes of SARS-CoV-2 [12]. Contrarily, linoleic acid-stabilized locked conformation of the spike protein shields essential immunogenic epitopes, enabling immune evasion [10].

In the present study, we established an in vitro model simulating the condition of high circulating levels of fatty acids, as found in patients with metabolic syndrome. Here, we observed an uptake of the fatty acids and consecutive triglyceride accumulation. Finally, we analyzed the effect of lipid accumulation on the replication of the SARS-CoV-2 Wuhan type or the variant of concern Delta in Calu-3 cells.

## 2. Materials and Methods

### 2.1. Cell Culture and Induction of Cellular Lipid Accumulation

Calu-3 cells were maintained in Minimal Essential Medium (MEM) containing 20% (*v/v*) inactivated fetal calf serum (FCS), 1 mM l-glutamine, 100 U/mL penicillin, and 100 μg/mL streptomycin and 1 mM sodium pyruvate.

The fatty acid oleate (Sigma, Steinheim, Germany) was complexed to bovine serum albumin (BSA) as described [13]. To induce cellular lipid accumulation, Calu-3 cells were incubated with oleate complexed to BSA for up to 72 h.

### 2.2. Lipid Analysis

Total triglycerides were extracted and quantified by a triglyceride determination kit (GPO-PAP) (Roche Diagnostics, Mannheim, Germany) as described [14]. Cellular lipid droplets were visualized by Oil Red O staining as described previously [13].

### 2.3. Analysis of mRNA Expression

Isolation of mRNA, reverse transcription, and quantitative real-time polymerase chain reaction (qRT-PCR) using specific sets of primers were performed as described [15]. Amplification of cDNA derived from actin was used for normalization of the data.

### 2.4. Viruses

The “Wuhan type” virus SARS-CoV-2_PR-1_, isolated from a 61-year-old patient, was amplified in Vero B4 cells as described in [16]. The virus strain SARS-CoV-2 Delta was obtained from Michael Schindler (University Hospital, Tübingen, Germany). The Delta variant (210601_INv) was isolated from throat swabs collected in May 2021 at the Institute for Medical Virology and Epidemiology of Viral Diseases, University Hospital Tübingen, from PCR-positive patients and generated as described in [17]. 

SARS-CoV-2 Viral titers of each variant were determined via an endpoint titration assay. For the generation of new virus stock, virus containing cell culture supernatant was harvested 72 h post infection (hpi) and passed through a 0.45 μm pore-size filter. All virus stocks were stored at −80 °C until further usage.

### 2.5. Infection Experiments

For infection experiments, cells were inoculated with SARS-CoV-2PR-1 (Wuhan type) or VoC Delta (multiplicity of infection (MOI): 2 × 10^−2^) for 1 h, washed and further treated with interventions. Next, 72 hpi, virus-containing cell culture supernatants were incubated for 10 min at 95 °C and used for qRT-PCR analysis. For titer determination of SARS-CoV-2 virus stocks, Calu-3 cells were infected with serial dilutions of the virus stock over 72 h. 

### 2.6. Determination of the Amount of Viral RNA Copies from Released Viruses by qRT-PCR

The amount of viral RNA copies in the virus-containing samples was quantified by real-time PCR Luna Universal Probe One-Step RT-PCR Kit from New England Biolabs (Cat: E3006L, Ipswich, MA, USA). This kit allows reverse transcription, cDNA synthesis, and PCR amplification to be carried out in a single step. Samples were analyzed by 7500 software v2.3 (Applied Biosystems, Waltham, MA, USA). PCR primers were designed and used as described previously in [18]. Thus, the polynucleotide sequence contains parts of the SARS-CoV-2 Envelope (E) and RNA-dependent RNA-polymerase (RdRp) genes and was used as standard for the determination of viral RNA copies in the experiments. The sequences of the used primers were: RdRp_forward (fwd): 5′-GTG-ARA-TGG-TCA-TGT-GTG-GCG-G-3′ and RdRp_reverse (rev) 5′-CAR-ATG-TTA-AAS-ACA-CTA-TTA-GCA-TA-C-3′. The probe was 5′--CAG-GTG-GAA-/ZEN/CCT-CAT-CAG-GAG-ATG-C -3′ (Label: FAM/IBFQ Iowa Black FQ). A dsDNA-polynucleotide sequence (Integrated DNA Technologies, Coralville, IA, USA) was used as a positive control: 5’-TAA-TAC-GAC-TCA-CTA-TAG-GGT-ATT-GAG-TGA-AAT-GGT-CAT-GTG-TGG-CGG-TTC-ACT-ATA-TGT-TAA-ACC-AGG-TGG-AAC-CTC-ATC-AGG-AGA-TGC-CAC-AAC-TGC-TTA-TGC-TAA-TAG-TGT-TTT-TAA-CAT-TTG-GAA-GAG-ACA-GGT-ACG-TTA-ATA-GTT-AAT-AGC-GTA-CTT-CTT-TTT-CTT-GCT-TTC-GTG-GTA-TTC-TTG-CTA-GTT-ACA-CTA-GCC-ATC-CTT-ACT-GCG-CTT-CGA-TTG-TGT-GCG-TAC-TGC-TGC-AAT-ATT-GTT-3′. Generating a series of dilutions (10^4^, 10^5^, 10^6^ and 10^7^ copies/mL) of this standard, the experiments were quantified using a standard curve to obtain absolute values of RNA copies in the sample.

### 2.7. Software and Statistics

Statistical analysis was carried out using GraphPad Prism Software version 6.01 (GraphPad Software, San Diego, CA, USA). Data are shown as the mean ± standard error of the mean (SEM). Datasets were compared through an analysis of the unpaired Student’s *t*-test or one-way ANOVA for multiple comparison tests when appropriate. A *p*-value < 0.05 was considered statistically significant.

## 3. Results

### 3.1. Effect of Oleate Treatment on Lipid Accumulation in Calu-3 Cells

**Figure 1:**
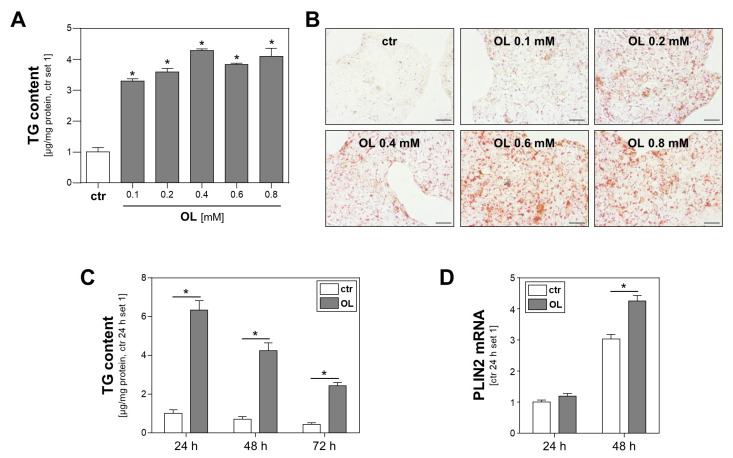
Effect of oleate treatment on lipid accumulation in Calu-3 cells. (**A**,**B**) Calu-3 cells were incubated with increasing oleate (OL) concentrations for 24 h. Cells treated with FFA-free BSA served as controls (ctr). (**A**) Triglyceride (TG) content normalized to cellular protein content (*: *p* < 0.05 compared to ctr). (**B**) Oil Red O staining; scale bars 50 µm. (**C**,**D**) Calu-3 cells were incubated with 0.6 mM OL or FFA-free BSA (ctr) for different time intervals, as indicated. (**C**) Triglyceride (TG) content normalized to cellular protein content and (**D**) PLIN2 mRNA levels analyzed by quantitative RT-PCR (*: *p* < 0.05).

Obesity and the metabolic syndrome, respectively, are characterized by hyperlipidemia. Free fatty acids reaching the circulation from adipose tissue are transported in the blood bound to albumin. We applied an in vitro model simulating these conditions to Calu-3 cells prior to infection with SARS-CoV-2. Here, oleic acid complexed with albumin was added to the cell culture medium. Control cells were incubated with the same amount of albumin but without fatty acids. Incubation with serial dilutions of the fatty acid oleate for 24 h induced a dose-dependent accumulation of intracellular triglycerides (Figure 1A) and cytosolic lipid droplets, as evidenced by Oil Red O staining (Figure 1B). Increased triglyceride levels slightly reduced over time, which was indicative of lipid combustion, but they persisted at a significantly increased level for at least 72 h (Figure 1C). Similarly, mRNA expression levels of perilipin-2 (PLIN2), a structural component of lipid droplets, were significantly higher in Calu-3 cells treated with oleate for 48 h compared to control cells (Figure 1D). In summary, these findings indicate the uptake of fatty acids and their storage in the form of lipid triglycerides.

### 3.2. Effect of Cellular Lipid Accumulation on the Replication of SARS-CoV-2

**Figure 2:**
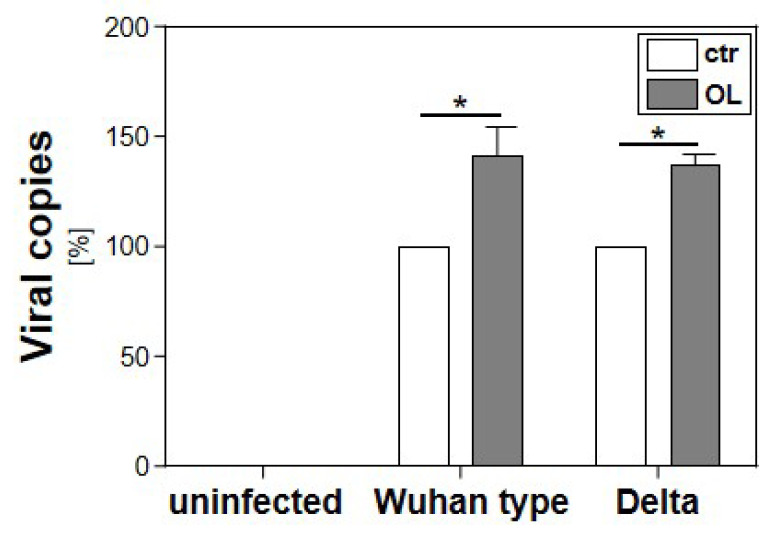
Effect of cellular lipid accumulation on the replication of SARS-CoV-2 Wuhan type or Delta. Calu-3 cells were incubated with 0.6 mM oleate for 5 h prior to infection with the SARS-CoV-2 Wuhan type or the VoC Delta (MOI of 2 × 10^−2^ for 1 h). Cell culture supernatants were harvested three days post infection. The virions were purified and analyzed by qRT-PCR. Bars show mean values of three independent experiments ± SEM (*: *p* < 0.05).

To assess the effect of cellular lipid accumulation on SARS-CoV-2 replication, Calu-3 cells were incubated with 0.6 mM oleate for 5 h prior to infection with the variants Wuhan type or Delta. Control cells were incubated with empty albumin. Three days post infection, cell culture supernatants were harvested, and virus production was analyzed by qRT-PCR. Preincubation with fatty acids and thus the induction of cellular lipid accumulation increased the replication of both SARS-CoV-2 variants by 50% (Figure 2).

## 4. Discussion

It has been described that patients with obesity or other components of the metabolic syndrome are mainly susceptible and more likely to exhibit severe side effects when affected by SARS-CoV-2. Still, the pathophysiological mechanisms of these phenomena are not completely understood.

Since hyperlipidemia is a major feature of obesity and the metabolic syndrome, we established a novel in vitro model to study the molecular mechanisms linking high fatty acids, such as those found in the circulation of patients with metabolic syndrome, and susceptibility to SARS-CoV-2. Here, we found that incubation with fatty acids caused significant and persistent triglyceride accumulation in Calu-3 cells. Importantly, we found that this cellular lipid accumulation significantly enhanced the replication of SARS-CoV-2 variants Wuhan and Delta. This led to the hypothesis that besides the potential direct viral effects on the host cell, the uptake of fatty acids from the circulation promotes SARS-CoV-2 replication, and thus the course of COVID-19. This pathophysiological mechanism may also contribute to a worse outcome in patients with components of the metabolic syndrome.

Importantly, in the present study, addition of oleic acid complexed with albumin did not alter the pH of the cell culture medium and did not inactivate SARS-CoV-2. Contrarily, in a study by Amruta et al., incubation with 6% acetic acid for 15 min effectively inactivated SARS-CoV-2 and completely inhibited virus replication [19]. Further studies are needed to elucidate whether varying pH has an influence on the effects of fatty acids on SARS-CoV-2.

Notably, increased plasma lipid levels have also been described in response to SARS-CoV-2 infection. Thomas et al. showed that COVID-19 patients have higher levels of free fatty acids in circulation compared to COVID-19-negative controls [20]. Yuan et al. analyzed the lipidomic serum profile of hospitalized COVID-19 patients over time and observed profound upregulation of triglyceride and diacylglycerol serum levels between day 0 and day 7 specimens [9]. The underlying molecular mechanisms by which SARS-CoV-2 affects circulating lipid levels in patients are not clearly known. However, it may be speculated that this phenomenon may be an additional enhancer of the course of disease, even in non-obese patients.

SARS-CoV-2 infection has been shown to directly influence the host lipid metabolism. An elegant study recently published by Farley et al. demonstrated that SARS-CoV-2 infection dramatically changed the host lipid metabolism by altering hundreds of lipid species [21]. Importantly, their study elucidated that viral propagation can be inhibited by glycerolipid biosynthesis blockers and that glycerolipid biosynthesis inhibition is effective against alpha, beta, gamma and delta virus types, illustrating the conserved dependency of SARS-CoV-2 on the host lipid metabolism [21]. From a more mechanistic point of view, a study by Dias et al. elucidated that SARS-CoV-2 infection led to increased expression of cluster of differentiation 36 (CD36, important for lipid uptake), peroxisome proliferator-activated receptor gamma (PPARγ) and sterol regulatory element-binding protein-1 (SREBP-1) (transcription factors involved in lipogenesis), and diacylglycerol O-acyltransferase 1 (DGAT-1, an enzyme involved in triacylglycerol synthesis) in primary human monocytes, indicating that cells were reprogrammed towards a lipogenic phenotype [8]. In line with this, viral nucleocapsid protein has also been shown to induce DGAT1 and DGAT2 expression in the human hepatoma cell line Huh7, as evidenced by a reporter gene assay [9]. Interestingly, in this study, DGAT1 and DGAT2 knockdown in human colonic Caco-2 cells and lung Calu-3 cells led to significantly reduced viral yields following infection compared to control-transfected cells [9]. In line with this, Dias et al. also showed that pharmacological inhibition of DGAT-1 reduced SARS-CoV-2 replication, inflammatory mediator production, and cell death [8]. A preliminary non-peer-reviewed publication by Ehrlich et al. demonstrated that human bronchial cells infected with SARS-CoV-2 and lung tissue from COVID-19 patients exhibit marked metabolic changes, i.e., changes in the pathways of endoplasmic reticulum stress, upregulation of glycolysis and dysregulation of the citric acid cycle, and upregulation of fatty acid and cholesterol synthesis [22]. Thus, not only uptake of fatty acids from the circulation, but also modulation of host lipid metabolism towards lipid accumulation and de novo lipogenesis by SARS-CoV-2 itself might substantially contribute to virus replication. However, further studies are needed to address this important issue.

In the present study, the effects of steatosis of the host cells on virus replication were comparable between the Wuhan and Delta strain. This is in line with a previous study that did not observe differences between different SARS-CoV-2 variants, including Wuhan and Delta, in terms of their impact on the host lipid metabolism [21]. Conversely, interference with the host cells lipid metabolism had a similar inhibitory effect on the replication of SARS-CoV-2 and its variants of concern [21,23].

Together, these findings indicate the conserved dependency of SARS-CoV-2 on host lipid metabolism and suggest interference with this dependency as a promising therapeutic strategy. The data presented here indicate elevated circulating lipid levels as a potential novel therapeutic target and potentially as prognostic marker for the disease courses during different phases of COVID-19.

## Data Availability

All data are included in the manuscript.

## References

[1] World Health Organization WHO Coronavirus Disease (COVID-19) Dashboard with Vaccination Data. https://covid19.who.int/?.

[2] Simonnet A., Chetboun M., Poissy J., Raverdy V., Noulette J., Duhamel A., Labreuche J., Mathieu D., Pattou F., Jourdain M. (2020). High Prevalence of Obesity in Severe Acute Respiratory Syndrome Coronavirus-2 (SARS-CoV-2) Requiring Invasive Mechanical Ventilation. Obesity.

[3] Barrasa H., Rello J., Tejada S., Martín A., Balziskueta G., Vinuesa C., Fernández-Miret B., Villagra A., Vallejo A., Sebastián A.S. (2020). SARS-CoV-2 in Spanish Intensive Care Units: Early experience with 15-day survival in Vitoria. Anaesth. Crit. Care Pain Med..

[4] Intensive Care National Audit and Research Center. https://www.icnarc.org/Our-Audit/Audits/Cmp/Reports.

[5] Peng Y.D., Meng K., Guan H.Q., Leng L., Zhu R.R., Wang B.Y., He M.A., Cheng L.X., Huang K., Zeng Q.T. (2020). Clinical characteristics and outcomes of 112 cardiovascular disease patients infected by 2019-nCoV. Zhonghua Xin Xue Guan Bing Za Zhi.

[6] Loosen S.H., Jensen B.-E.O., Tanislav C., Luedde T., Roderburg C., Kostev K. (2022). Obesity and lipid metabolism disorders determine the risk for development of long COVID syndrome: A cross-sectional study from 50,402 COVID-19 patients. Infection.

[7] Nardacci R., Colavita F., Castilletti C., Lapa D., Matusali G., Meschi S., Del Nonno F., Colombo D., Capobianchi M.R., Zumla A. (2021). Evidences for lipid involvement in SARS-CoV-2 cytopathogenesis. Cell Death Dis..

[8] Dias S.S.G., Soares V.C., Ferreira A.C., Sacramento C.Q., Fintelman-Rodrigues N., Temerozo J.R., Teixeira L., da Silva M.A.N., Barreto E., Mattos M. (2020). Lipid droplets fuel SARS-CoV-2 replication and production of inflammatory mediators. PLoS Pathog..

[9] Yuan S., Yan B., Cao J., Ye Z.-W., Liang R., Tang K., Luo C., Cai J., Chu H., Chung T.W.-H. (2021). SARS-CoV-2 exploits host DGAT and ADRP for efficient replication. Cell Discov..

[10] Toelzer C., Gupta K., Berger I., Schaffitzel C. (2023). Cryo-EM reveals binding of linoleic acid to SARS-CoV-2 spike glycoprotein, suggesting an antiviral treatment strategy. Acta Crystallogr. Sect. D Struct. Biol..

[11] Staufer O., Gupta K., Bücher J.E.H., Kohler F., Sigl C., Singh G., Vasileiou K., Relimpio A.Y., Macher M., Fabritz S. (2022). Synthetic virions reveal fatty acid-coupled adaptive immunogenicity of SARS-CoV-2 spike glycoprotein. Nat. Commun..

[12] Doaei S., Gholami S., Rastgoo S., Gholamalizadeh M., Bourbour F., Bagheri S.E., Samipoor F., Akbari M.E., Shadnoush M., Ghorat F. (2021). The effect of omega-3 fatty acid supplementation on clinical and biochemical parameters of critically ill patients with COVID-19: A randomized clinical trial. J. Transl. Med..

[13] Wobser H., Dorn C., Weiss T., Amann T., Bollheimer C., Büttner R., Schölmerich J., Hellerbrand C. (2009). Lipid accumulation in hepatocytes induces fibrogenic activation of hepatic stellate cells. Cell Res..

[14] Mahli A., Seitz T., Freese K., Frank J., Weiskirchen R., Abdel-Tawab M., Behnam D., Hellerbrand C. (2019). Therapeutic Application of Micellar Solubilized Xanthohumol in a Western-Type Diet-Induced Mouse Model of Obesity, Diabetes and Non-Alcoholic Fatty Liver Disease. Cells.

[15] Seitz T., Freese K., Dietrich P., Thasler W.E., Bosserhoff A., Hellerbrand C. (2020). Fibroblast Growth Factor 9 is expressed by activated hepatic stellate cells and promotes progression of hepatocellular carcinoma. Sci. Rep..

[16] Große M., Ruetalo N., Layer M., Hu D., Businger R., Rheber S., Setz C., Rauch P., Auth J., Fröba M. (2021). Quinine Inhibits Infection of Human Cell Lines with SARS-CoV-2. Viruses.

[17] Fröba M., Große M., Setz C., Rauch P., Auth J., Spanaus L., Münch J., Ruetalo N., Schindler M., Morokutti-Kurz M. (2021). Iota-Carrageenan Inhibits Replication of SARS-CoV-2 and the Respective Variants of Concern Alpha, Beta, Gamma and Delta. Int. J. Mol. Sci..

[18] Corman V.M., Landt O., Kaiser M., Molenkamp R., Meijer A., Chu D.K.W., Bleicker T., Brünink S., Schneider J., Schmidt M.L. (2020). Detection of 2019 novel coronavirus (2019-nCoV) by real-time RT-PCR. Eurosurveillance.

[19] Amruta N., Maness N.J., Gressett T.E., Tsuchiya Y., Kishi M., Bix G. (2023). Effect of acetic acid inactivation of SARS-CoV-2. PLoS ONE.

[20] Thomas T., Stefanoni D., Reisz J.A., Nemkov T., Bertolone L., Francis R.O., Hudson K.E., Zimring J.C., Hansen K.C., Hod E.A. (2020). COVID-19 infection alters kynurenine and fatty acid metabolism, correlating with IL-6 levels and renal status. JCI Insight.

[21] Farley S.E., Kyle J.E., Leier H.C., Bramer L.M., Weinstein J.B., Bates T.A., Lee J.-Y., Metz T.O., Schultz C., Tafesse F.G. (2022). A global lipid map reveals host dependency factors conserved across SARS-CoV-2 variants. Nat. Commun..

[22] Ehrlich A., Uhl S., Ioannidis K., Hofree M., tenOever B.R., Nahmias Y. (2020). The SARS-CoV-2 Transcriptional Metabolic Signature in Lung Epithelium. SSRN Electron. J..

[23] Aliyari S.R., Ghaffari A.A., Pernet O., Parvatiyar K., Wang Y., Gerami H., Tong A.-J., Vergnes L., Takallou A., Zhang A. (2022). Suppressing fatty acid synthase by type I interferon and chemical inhibitors as a broad spectrum anti-viral strategy against SARS-CoV-2. Acta Pharm. Sin. B.

